# ALK1Fc Suppresses the Human Prostate Cancer Growth in *in Vitro* and *in Vivo* Preclinical Models

**DOI:** 10.3389/fcell.2017.00104

**Published:** 2017-12-05

**Authors:** Letizia Astrologo, Eugenio Zoni, Sofia Karkampouna, Peter C. Gray, Irena Klima, Joël Grosjean, Marie J. Goumans, Lukas J. A. C. Hawinkels, Gabri van der Pluijm, Martin Spahn, George N. Thalmann, Peter ten Dijke, Marianna Kruithof-de Julio

**Affiliations:** ^1^Department of Urology and Department for BioMedical Research, Urology Research Laboratory, University of Bern, Bern, Switzerland; ^2^Department of Urology, Leiden University Medical Centre, Leiden, Netherlands; ^3^Department of Molecular Cell Biology, Cancer Genomics Center, Leiden University Medical Centre, Leiden, Netherlands; ^4^Clayton Foundation Laboratories for Peptide Biology, Salk Institute for Biological Studies, La Jolla, CA, United States; ^5^Department of Gastroenterology-Hepatology, Leiden University Medical Centre, Leiden, Netherlands

**Keywords:** BMP9, ALK1, ALK2, ALK1Fc, NOTCH, prostate cancer

## Abstract

Prostate cancer is the second most common cancer in men and lethality is normally associated with the consequences of metastasis rather than the primary tumor. Therefore, targeting the molecular pathways that underlie dissemination of primary tumor cells and the formation of metastases has a great clinical value. Bone morphogenetic proteins (BMPs) play a critical role in tumor progression and this study focuses on the role of BMP9- *A*ctivin receptor-*L*ike *K*inase 1 and 2 (ALK1 and ALK2) axis in prostate cancer. In order to study the effect of BMP9 *in vitro* and *in vivo* on cancer cells and tumor growth, we used a soluble chimeric protein consisting of the ALK1 extracellular domain (ECD) fused to human Fc (ALK1Fc) that prevents binding of BMP9 to its cell surface receptors and thereby blocks its ability to activate downstream signaling. ALK1Fc sequesters BMP9 and the closely related BMP10 while preserving the activation of ALK1 and ALK2 through other ligands. We show that ALK1Fc acts *in vitro* to decrease BMP9-mediated signaling and proliferation of prostate cancer cells with tumor initiating and metastatic potential. In line with these observations, we demonstrate that ALK1Fc also reduces tumor cell proliferation and tumor growth *in vivo* in an orthotopic transplantation model, as well as in the human patient derived xenograft BM18. Furthermore, we also provide evidence for crosstalk between BMP9 and NOTCH and find that ALK1Fc inhibits NOTCH signaling in human prostate cancer cells and blocks the induction of the NOTCH target Aldehyde dehydrogenase member ALDH1A1, which is a clinically relevant marker associated with poor survival and advanced-stage prostate cancer. Our study provides the first demonstration that ALK1Fc inhibits prostate cancer progression, identifying BMP9 as a putative therapeutic target and ALK1Fc as a potential therapy. Altogether, these findings support the validity of ongoing clinical development of drugs blocking ALK1 and ALK2 receptor activity.

## Introduction

Prostate cancer is the second most common cancer in men worldwide (Jemal et al., 2010). Currently prostate cancer, when still in its first phase of androgen dependency, can be successfully treated surgically. However, if the tumor becomes androgen independent, therapy is no longer possible and lethality is almost invariably due to the consequences of metastasis. Therefore, understanding the molecular pathways that underlie the emergence and spread of metastases from primary tumors is of great biological and clinical value.

Expression of several BMPs has been examined in prostatic tissue with benign prostatic hyperplasia (BPH), non-metastatic and metastatic prostatic adenocarcinoma and has been associated with cancer aggressiveness (Ye et al., 2007; Ye and Jiang, 2016). Among the BMPs, BMP9 is one of the most recently identified (Song et al., 1995). Little is known about the roles of BMP9 and its cell surface signaling receptors, ALK1 and ALK2, in prostate cancer and particularly in androgen independent and metastatic prostate cancer. Current research has not only attributed a tumor-promoting role to BMP9 (Herrera et al., 2009, 2013; Li et al., 2014) but also tumor suppressing properties (Ye et al., 2008; Wang et al., 2011; Olsen et al., 2014) in different types of cancer, including prostate cancer.

Previous studies have highlighted the role of ALK1, which is predominantly expressed by endothelial cells (van Meeteren et al., 2012), as key regulator of angiogenesis in normal tissue and in tumors (Hawinkels et al., 2013; Bendell et al., 2014). BMP9 and BMP10 are high affinity ligands for ALK1, while BMP9 signals through the BMP type I receptor ALK2 (David et al., 2007; Herrera et al., 2009; Bragdon et al., 2011). Binding of BMP9/BMP10 to ALK1/ALK2 results in phosphorylation and activation of downstream effectors SMAD1 and/or SMAD5 (David et al., 2007; Scharpfenecker et al., 2007; Herrera et al., 2009). BMP9 promotes human epithelial ovarian cancer and human immortalized ovarian surface epithelial cell proliferation through ALK2/SMAD1/SMAD4 pathway (Herrera et al., 2009). Similarly, BMP9 stimulates proliferation of liver cancer cells (Herrera et al., 2013) and osteosarcoma growth (Li et al., 2014).

Several studies have highlighted the role of BMP9/ALK1 in blood vessel formation, outlining its critical involvement in pathological and tumor angiogenesis (Urness et al., 2000; Cunha and Pietras, 2011). Interestingly, alterations of signal transduction pathways that are important for blood vessel formation, such as the NOTCH pathway, have also been associated with arterio-venous malformations (Gale et al., 2004; Krebs et al., 2004). Recently, BMP9 and BMP10 signaling were linked to NOTCH signaling, one of the major pathways involved in prostate cancer development, progression and bone metastasis (Carvalho et al., 2014; Kron et al., 2017; Zhang et al., 2017). Expression profiling studies have shown that members of the NOTCH pathway are characteristic of high-grade (Gleason 4 + 4 = 8) micro-dissected prostate cancer cells compared to low-grade (Gleason 3 + 3 = 6) (Ross et al., 2011). Moreover, inhibition of NOTCH1 reduces prostate cancer cell growth, migration and invasion (Wang et al., 2010). Interestingly, the NOTCH signaling indirectly activates the enzymatic activity of ALDH1A1, a well-known marker of prostate cancer stem cells (Ginestier et al., 2007; Li et al., 2010; Le Magnen et al., 2013; Zhao et al., 2014), which are thought to be responsible for tumor recurrence, metastasis and cancer related death (Moltzahn and Thalmann, 2013).

In order to understand the role of BMP9 in prostate cancer progression, we employed the soluble chimeric protein ALK1Fc (ACE-041) (Seehra et al., 2009) which binds BMP9 and BMP10 with high affinity and blocks their signaling via ALK1 and ALK2 receptors by acting as a ligand trap (Cunha et al., 2010; Mitchell et al., 2010). Phase I clinical trials have been completed using ALK1Fc as anti-angiogenesis therapy in myeloma (clinicaltrials.gov identifier NCT00996957). Here we show that ALK1Fc reduces BMP9 signaling and decreases proliferation of highly metastatic human prostate cancer cells *in vitro*. We further demonstrate that ALK1Fc impairs tumor angiogenesis, affects tumor cell proliferation and reduces tumor growth *in vivo*. Taken together these data suggest BMP9 as a possible therapeutic target in prostate cancer and provide a new rationale for ongoing clinical development of drugs blocking BMP9 signaling via ALK1 and ALK2.

## Materials and methods

### Cell line and culture conditions

The human osteotropic prostate cancer cell line PC-3M-Pro4-Luc2 (Kroon et al., 2014; Zoni et al., 2015, 2017) was maintained in DMEM supplemented with 10% FCII, 0.8 mg/ml Neomycin (Santacruz, Dallas, USA) and 1% Penicillin-Streptomycin (Life Technologies, Carlsbad, USA).

### Recombinant proteins and chemical inhibitors

ALK1Fc (de Vinuesa et al., 2016) is a fusion protein comprised of the extracellular domain (ECD) of human ALK1 fused to the Fc region of IgG and was obtained from Acceleron Pharma, Cambridge, USA. As a control we used either the Fc domain of IgG_1_ (MOPC-21; Bio Express, West Lebanon NH) or normal goat IgG from R&D System.

Recombinant human BMP9 was obtained from R&D System, whereas the chemical inhibitor LDN193189 was purchased from Axon Medchem. The final concentration for the *in vitro* experiments was 1 nM for BMP9 and 120 nM for LDN193189.

### Lentiviral-mediated RNA interference of NOTCH1

shRNAi for NOTCH1 (TRC000000350253, TRC000000350330, TRC0000003361, TRC0000003360) were obtained from Sigma MISSION library and used for lentiviral vector production and transduction as described previously (Zoni et al., 2017). Scramble shRNA (NT; SHC002, Sigma) was used as control. The transduced cells underwent puromycin selection and used for further experiments as described below. The experiments were carried out in accordance with standard biosecurity procedures.

### Luciferase reporter assays and constructs

PC-3MPro4 cells were seeded at density of 50,000 cells in 500 μL medium in a 24-well plate. Transient transfection of reporter constructs was performed with Lipofectamine2000 (Life Technologies) according to the manufacturer's protocol. For each well, 100 ng of NICD-ff-luciferase, 10 ng CAGGS-Renilla luciferase, 100 ng BRE renilla (Korchynskyi and ten Dijke, 2002) and 100 ng BREluc/well were transfected. After 24 h, medium was replaced and cells were treated with BMP9 for 24 h. The *Firefly* luciferase and *Renilla* luciferase levels in the lysates were measured using Dual Luciferase Assay (Promega, Madison, USA).

### RNA isolation and real-time Q-PCR

Total RNA was isolated from PC-3M-Pro4-Luc2 cells with Trizol Reagent (Invitrogen, Waltham, USA) and cDNA was synthesized by reverse transcription (Promega, Madison, USA) according to the manufacturer's protocol. qRT-PCR was performed with Biorad CFX96 system (Biorad, Veenendaal, The Netherlands). Gene expression was normalized to *GAPDH* or β*-actin*. Total RNA from frozen section (5 μm) was isolated with Qiagen Mini Isolation kit (Venlo, The Netherlands) according to the manufacturer's protocol. Primer sequences are listed in Supplementary Table I.

### MTS assay

Cells were seeded at density of 2,000 cells/well in low serum condition (0.3% FCII), treated with ALK1Fc or Control-Fc (CFc) (10 μg/ml, Acceleron, USA) and allowed to grow for 24, 48, 72, and 96 h. After incubation, 20 μl of 3-(4,5 dimethylthiazol- 2- yl)- 5 -(3 -carboxymethoxyphenyl)- 2 -(4 -sulfophenyl)- 2 H-tetrazolium (MTS) was added and mitochondrial activity was measured after 2 h incubation at 37°C. MTS absorbance values are positively proportional to total number of metabolically active cells providing an indirect correlation with cell proliferation rate (CellTiter96 Aqueous Non-radioactive Cell proliferation assay, Promega) (Berridge et al., 2005).

### Animals

Male 6–8 week-old athymic nude (Balb/c *nu/nu*) or CB17 SCID mice, purchased from Charles River (L'Arbresle, France), were used in all *in vivo* experiments. Mice were housed in individual ventilated cages under sterile condition, and sterile food and water were provided *ad libitum*. Animal experiments were approved by the local committee for animal health ethics and research of Leiden University (DEC #11246) and Canton of Bern, Switzerland (Permit Number: BE55/16), and carried out in accordance with European Communities Council Directive 86/609/EEC and Swiss Guidelines for the Care and Use of Laboratory Animals.

### Orthotopic prostate transplantation and ALK1Fc treatment

25,000 PC-3M-Pro4-Luc2 cells (10 μl final volume) were injected in the dorsal prostate lobe of anesthetized male nude mice. In brief: After anesthetizing the mice with isoflurane, each mouse was placed on its back and a small incision was made along the lower midline of the peritoneum for about 1 cm. The prostate dorsal lobes were exteriorized and stabilized gently. A 30-gauge needle attached to a 1-cc syringe was inserted into the right dorsal lobe of the prostate. 10 μl of the cell suspension was slowly injected. A well-localized bleb indicates a successful injection. After retracting the needle, a Q-tip was placed over the injection site for about 1 min to prevent bleeding and spillage of material. The prostate was then returned to the peritoneum and the abdominal wall and skin layer was sutured. After establishment of the primary tumor, at 10 days after the orthotopic transplantation, mice were intraperitoneally injected with Control-Fc (CFc) or ALK1Fc compounds (10 mg/kg) twice per week. Administration of compounds was performed for 4 weeks. After the experimental periods, mice were injected with hypoxia probe (6 mg/kg, Burlington, Massachusetts, USA) and lectin-Tomato (1 mg/kg, Vector Laboratories, USA) intravenously prior to perfusion and sacrificed according to our mouse protocol. Tumors were dissected and processed for further histomorphological analysis as described below.

### Subcutaneously BM18 transplantation and ALK1Fc treatment

BM18 xenografts were transplanted subcutaneously in CB17 SCID mice anesthetized with a cocktail of medetomidin (1 mg/kg body weight), midazolam (10 mg/kg) and fentanyl (0.1 mg/kg) (Schwaninger et al., 2007). After 1 week, the animals were intraperitoneally injected with ALK1Fc or IgG at the dose of 10 mg/kg once a week, for 5 weeks. Every week the tumors were measured with the caliper and finally dissected and fixed in 4% paraformaldehyde (PFA) for paraffin embedding and hematoxylin and eosin staining.

### Whole body bioluminescent imaging (BLI)

Tumor growth from orthotopic injection was monitored weekly by whole body bioluminescent imaging (BLI) using an intensified-charge-coupled device (I-CCD) video camera of the *in vivo* Imaging System (IVIS100, Xenogen/Perkin Elmer, Alameda, CA, USA) as described previously (Buijs et al., 2007; van den Hoogen et al., 2010). In the orthotopic transplantation experiment the newer IVIS Lumina II (Xenogen/Perkin Elmer, Alameda, CA, USA) was used for BLI measurements. Mice were anesthetized using isoflurane and injected intraperitoneally with 2 mg D-luciferin (Per bio Science Nederland B.V., Etten-Leur, the Netherlands). Analyses for each metastatic site were performed after definition of the region of interest and quantified with Living Image 4.2 (Caliper Life Sciences, Teralfene, Belgium). Values are expressed as relative light units (RLU) in photons/s.

### Immunofluorescence

Immunofluorescence staining was performed on 5-μm paraffin embedded sections. For antigen retrieval, sections were boiled in antigen unmasking solution (Vector Labs, Peterborough, UK) and stained with anti- pH3 (Millipore), cleaved CASP3 (Cell Signaling), CD31 (Sigma) or ALDH1A1 (Abcam) antibodies. Sections were blocked with 1% bovine serum albumin (BSA)-PBS-0.1% v/v Tween-20 and incubated with primary antibodies diluted in the blocking solution, overnight at 4°C. Sections were then incubated with secondary antibodies labeled with Alexa Fluor 488, 555, or 647 (Invitrogen/Molecular Probes, Waltham, USA) at 1:250 in PBS-0.1% Tween-20. Nuclei were visualized by TO-PRO3 (Invitrogen/Molecular Probes, 1:1000 diluted in PBS-0.1% Tween-20) (Karkampouna et al., 2014).

### Western immunoblotting

Cell lysates were prepared using RIPA buffer (Thermo Scientific) and protein concentrations were quantified according to manufacturer's protocol (Thermo Scientific). Proteins (20 μg per sample) were separated by 15% SDS-PAGE followed by transfer to a blotting membrane. The membrane was blocked with 5% Milk, dissolved in PBS-Tween, for 1 h at room temperature. The membrane was incubated with 1:1,000 primary antibody (anti-NOTCH1, Cell Signaling, catalog number 3608) at 4°C overnight. Subsequently, the membrane was incubated with 1:10,000 secondary horseradish peroxidase (HRP) antibody. All antibodies were dissolved in PBS-Tween. Chemiluminescence was used to visualize the bands.

### Clonogenic assay

Clonogenic assay was performed in 6 well plate. 100 cells were seeded in 2 mL of medium and incubated at 37°C in presence of 5% CO_2_ for 2 weeks. Plates were washed with PBS and cells fixed for 5 min with a solution of 4% PFA. Colonies were stained with 0.1% crystal violet (Sigma-Aldrich, The Netherlands) and plates were imaged before processing the data with ImageJ software (Franken et al., 2006; Rafehi et al., 2011; Guzman et al., 2014).

### Prostate cancer dataset analysis

The Taylor MSKCC prostate dataset was queried for BMP9 and ALK1 expression in prostate cancer patients through the online biomarker validation tool SurvExpress (http://bioinformatica.mty.itesm.mx:8080/Biomatec/SurvivaX.jsp). The data are censored as “recurrence month,” and the risk groups are defined estimating a prognostic index by the Cox model algorithm (Aguirre-Gamboa et al., 2013).

R2: Genomics Analysis and Visualization Platform (http://r2.amc.nl) was used to investigate the ALK2, JAG1 and NOTCH1 expression in benign (*n* = 48) vs. tumor (*n* = 47) tissues using the GEO accession number GSE29079 dataset (Borno et al., 2012).

### Statistical analysis

Statistical analysis was performed with GraphPad Prism 6.0 (GraphPad software) using *t*-test or ANOVA for comparison between more groups. Data is presented as mean ± SEM. *P*-values < 0.05 were considered to be statistically significant (^*^P < 0.05, ^**^P < 0.01, ^***^P < 0.001).

## Results

### High BMP9 and ALK1 correlate with recurrence in prostate cancer patients

**Figure 1:**
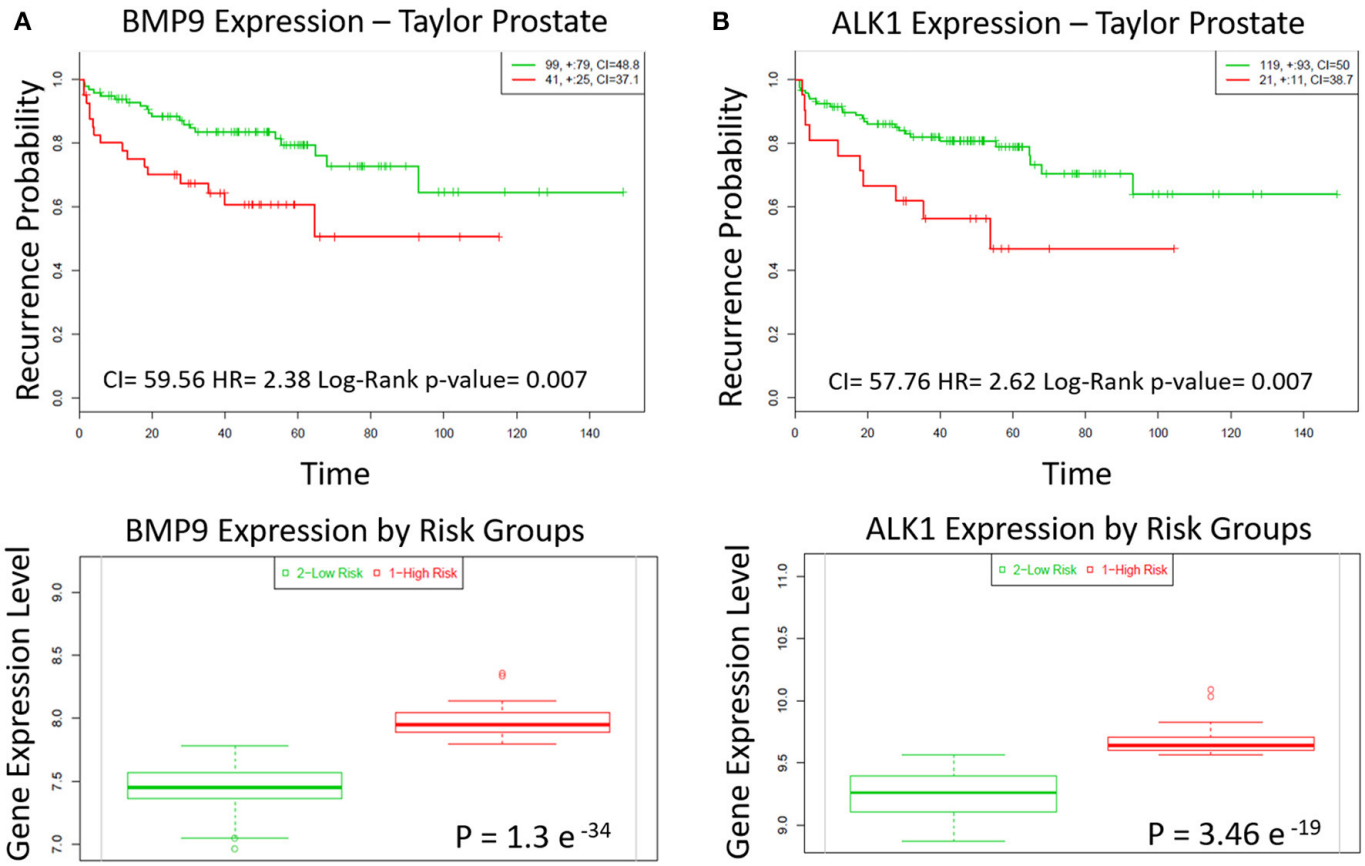
BMP9 and ALK1 correlate with poor patient prognosis. **(A,B)** Top panels: Kaplan-Meier survival curves of censored Cox analysis in Taylor-MSKCC prostate database stratified by maximized BMP9 and ALK1 expression risk groups. Subjects with a higher expression display earlier biochemical recurrence than subjects with a lower risk (Bovelstad and Borgan, 2011). Red, high expression; Green, low expression. CI, Concordance Index; HR, Risk Group Hazard Ratio. Bottom panels: BMP9 and ALK1 expression levels stratified by risk groups. Red, high Risk; Green, low risk.

The role of BMP9 in cancer development and progression is still controversial. We analyzed a Taylor MSKCC Prostate dataset (GSE21032) through the online tool SurvExpress to assess how the expression of BMP9 and ALK1 is related with recurrence in prostate cancer. We found that the group of patients with high expression of BMP9 and ALK1 has higher probability to encounter biochemical recurrence than the group with lower levels of BMP9 and ALK1 (Figures 1A,B top). The SurvExpress tool also defines “high-” and “low-” risk group of patients based on the risk prognosis calculated as described in the Material and Methods. According to that definition, we also found that the high-risk group of patients had higher expression of BMP9 and ALK1 than lower risk patients (Figures 1A,B bottom). Therefore, we explored whether inhibition of BMP9 signaling in mouse models of prostate cancer interferes with tumor growth.

### ALK1Fc reduces primary prostate tumor burden *in vivo*

**Figure 2:**
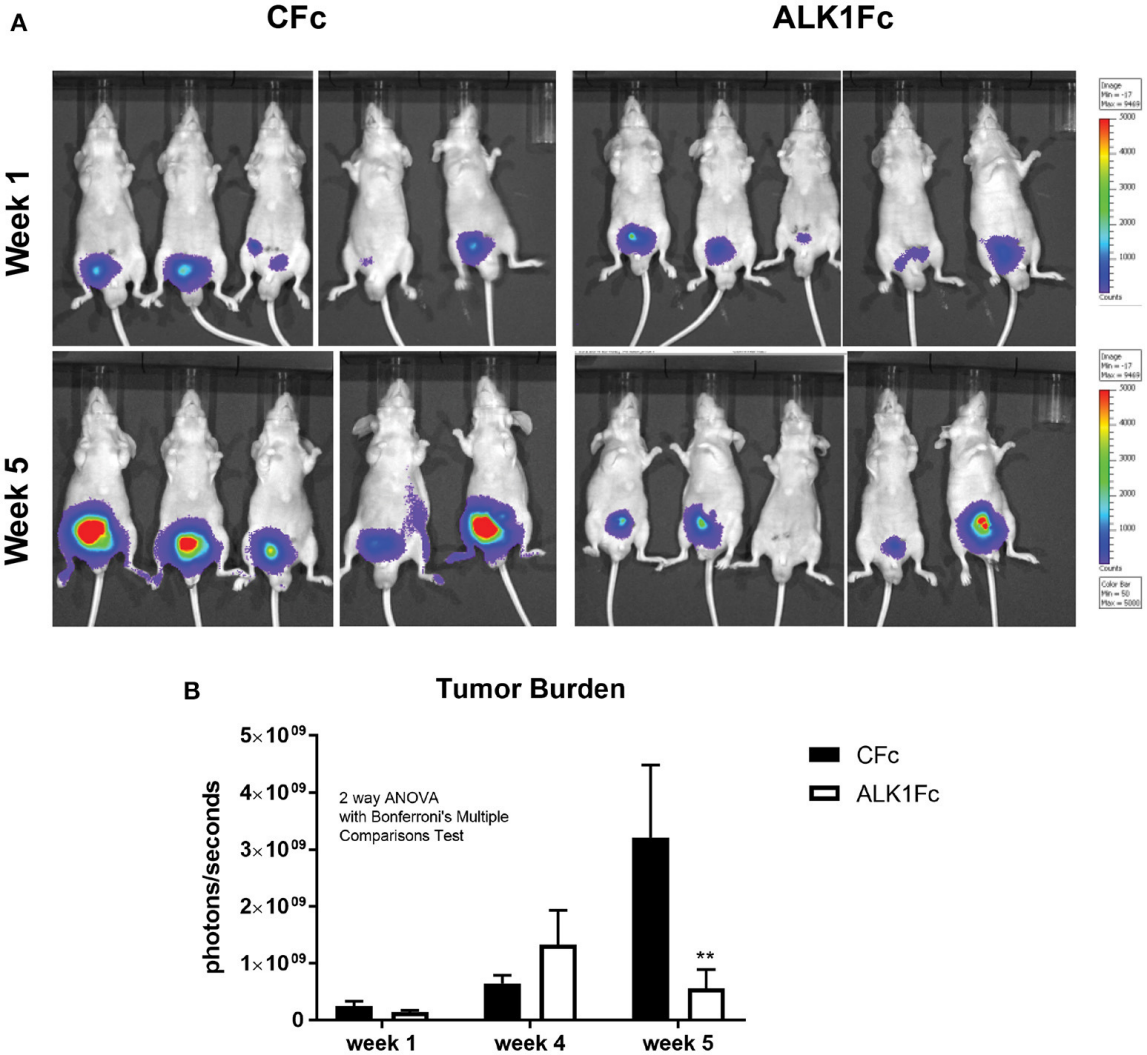
ALK1FC reduces primary prostate tumor burden. **(A)** PC-3M-Pro4-Luc2 cells were orthotopically injected in the dorsal lobe of prostate glands of nude mice (*n* = 15 per group). Detection of primary tumor burden was observed at 2 weeks after injection, with the time point designated as “week 1” at the start of treatment with ALK1Fc or CFc. Representative examples of bioluminescent images of tumor burden at the start of treatment with ALK1Fc/CFc (week 1) and at the end point (week 5). **(B)** Quantification of bioluminescent signal (photons/sec) in mice treated with either CFc (*n* = 14) or ALK1Fc (*n* = 15) for 5 weeks. Error bars indicate ± SEM. ^**^P-value < 0.01.

To investigate the role of BMP9 in prostate cancer progression, the BMP9 ligand trap ALK1Fc was administered in an orthotopic mouse model of prostate cancer. Primary prostate tumor growth was induced by intra-prostatic inoculation of human prostate cancer PC-3M-Pro4-Luc2 cells in Balb/c nude mice and tumor progression was followed by bioluminescence imaging (BLI) (Kroon et al., 2014) (Figure 2A). Based on the BLI signal the mice were randomized in two treatment groups: ALK1Fc or control (C) Fc (*n* = 15 per group). The recombinant proteins were injected twice weekly and tumor imaging and body weights were monitored weekly for 5 weeks (Supplementary Figure 1). Tumor burden was quantitatively assessed for each animal during the course of treatment. The group of animals that received ALK1Fc exhibited smaller tumor size compared to the animals that received CFc based on bioluminescence quantification (Figure 2B, *p* < 0.01).

### ALK1Fc reduces cell proliferation in the primary prostate tumor

**Figure 3:**
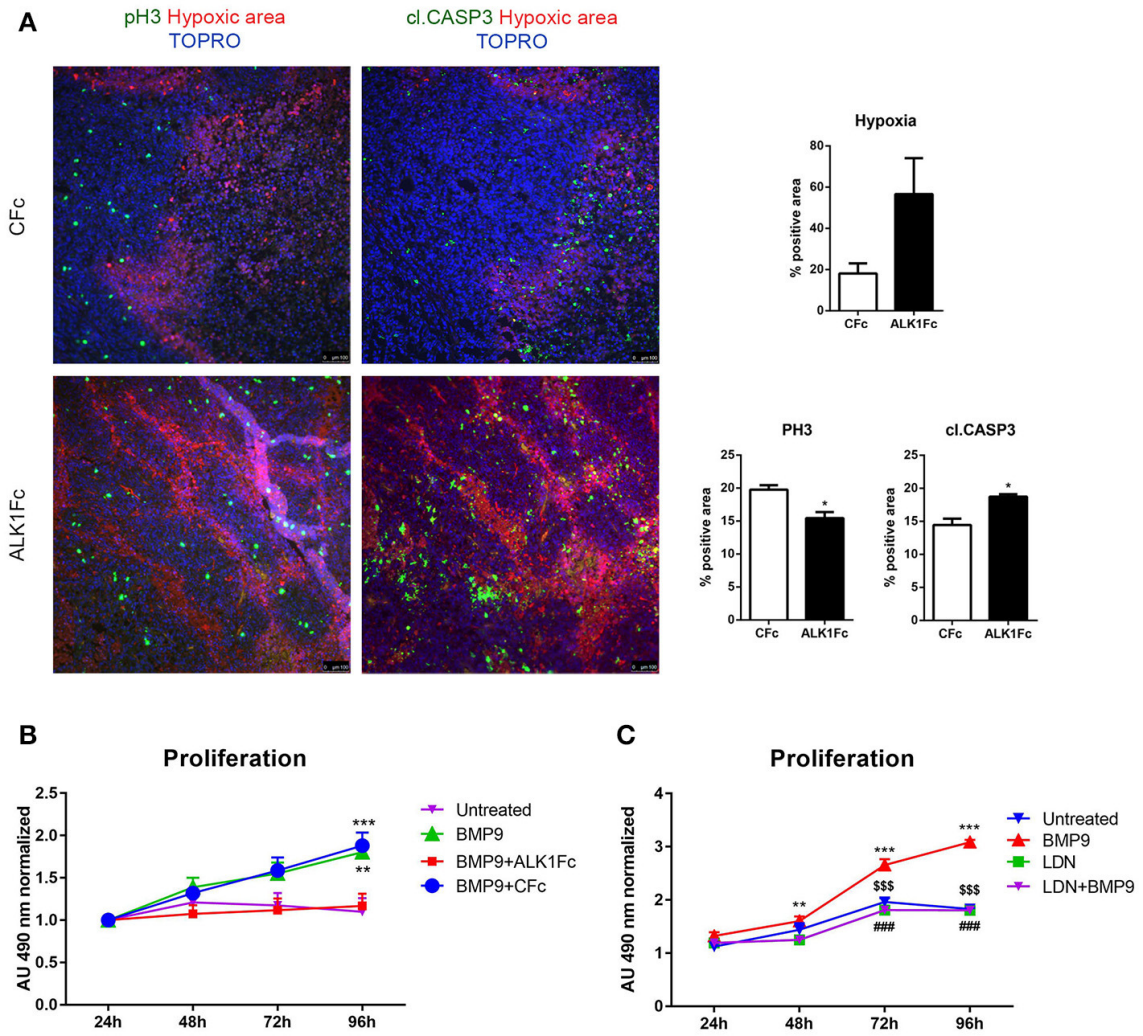
ALK1Fc increases hypoxia and apoptosis and decreases cell proliferation *in vivo*. **(A)** Left panels: Representative images of hypoxia immunofluorescence staining (red) in primary prostate tumor samples after 5 weeks of treatment with either ALK1Fc or CFc. Hypoxia probe was injected prior to sacrifice and was detected by a specific fluorescent antibody. Immunofluorescence images for colocalization of apoptotic or proliferating cells in hypoxic/normoxic area within the prostate tumor area in ALK1Fc and CFc treated animals. pH3: PhosphoHistone 3 proliferation marker (green); cleaved caspase 3 apoptosis marker (green); Hypoxic probe-antibody: hypoxic area (red); TOPRO: nuclear dye (blue). Right graphs: Quantification of hypoxia, pH3 and cl.CASP3 positive area in all tumor samples of each group (*n* = 6 for CFc, *n* = 7 for ALK1Fc). **(B)** MTS assay (24, 48, 72, 96 h) was performed in PC-3M-Pro4-Luc2 cells stimulated with recombinant BMP9 (1 nM), BMP9 (1 nM) + ALK1Fc (10 μg/ml), or BMP9 (1 nM) + CFc (10 μg/ml). Accumulation of MTS was measured based on absorbance at 490 nm. Values are normalized to the basal measurements at 24 h after cell seeding and treatments. Graph represents values for three independent experiments (*n* = 3). Error bars indicate ± SEM. ^**^P-value < 0.01 BMP9 vs. Untreated and ^***^P-value < 0.001 BMP9+CFc vs. Untreated. **(C)** MTS assay (24, 48, 72, and 96 h) performed in PC-3M-Pro4-Luc2 cells seeded at low density in 96-well plates and treated with BMP9 (1 nM), LDN (BMP type I receptor inhibitor LDN193189, 120 nM) or LDN+BMP9. (*n* = 2). Values are normalized to the basal measurements at the time of cell seeding and treatments. Error bars indicate SEM. ^**^P-value < 0.01 BMP9 vs. LDN and BMP9 vs. LDN+-BMP9; ^***^P-value < 0.001 BMP9 vs. Untreated; ^$$$^BMP9 vs. LDN; ^###^BMP9 vs. LDN+BMP9; ^*^P-value < 0.05.

The degree of tumor angiogenesis is critical for progressive tumor growth beyond a few mm^3^ in size. Intravital lectin perfusion was used to map the perfused elements of the tumor vasculature in mice. Fluorescent-conjugated lectin (lectin-Tomato) was visualized in tumor tissue sections and quantified. We observed a trend in decreased vascular density, indicated by the overall lectin presence, in the tumors treated with ALK1Fc compared to the CFc group (Supplementary Figures 2A,B). We evaluated the presence of endothelial cells in tumor sections by CD31 immunofluorescence. A trend of decreased CD31 expression was also observed after treatment with ALK1Fc suggesting fewer endothelial cells and vessels (Supplementary Figures 2C,D). Hypoxia is an important component of angiogenesis and critical for tumor formation. A hypoxia-induced probe was injected in tumor bearing mice just prior to sacrifice and the hypoxic areas within the tumors were visualized after tumor resection (Figure 3A; left panels). Although hypoxic areas were found in both treatment groups, the overall amount of hypoxia seemed to be higher in ALK1Fc-treated mice relative to the CFc-treated mice (Figure 3A; right graph, *p* = 0.050). We assessed the presence of cell proliferation and cell death in these tumors by immunofluorescence for the mitosis marker phosphorylated histone 3 (PH3) and the apoptosis marker cleaved caspase 3 (CASP3), respectively. Dividing PH3 positive cells are predominantly located in normoxic areas (Figure 3A; left panel). Quantification of immunofluorescence signal shows that the number of dividing cells is lower in the ALK1Fc-treated animals (Figure 3A; right graph *p* < 0.05). Detection of apoptotic cells (Caspase-3 positive) is higher in the ALK1Fc-treated tumors (Figure 3A; right graph *p* < 0.05) and occurs mostly, but not exclusively, in hypoxic areas (Figure 3A; left panel), suggesting a correlation between the hypoxia and tumor cell death.

### ALK1Fc decreases proliferation of human prostate cancer cells *in vitro*

To investigate how ALK1Fc can decrease tumor growth, we studied the effect of ALK1Fc on prostate cancer cells. We measured the mRNA levels of the BMP9 type I receptors ALK1 and ALK2 in the PC-3M-Pro4-Luc2 (Kroon et al., 2014) human prostate cancer cell line and tested their response to BMP9. Consistent with a previous report in highly metastatic PC-3 and PC-3M prostate cancer cells (Craft et al., 2007), qRT-PCR analysis in osteotropic PC-3M-Pro4-Luc2 cells revealed undetectable levels of ALK1 but measurable levels of ALK2 (Supplementary Figure 3A). Treatment with BMP9 showed a dose-dependent induction of BRE-*Renilla* luciferase (luc) activity in PC-3M-Pro4 cells (*p*-value = 0.005 and 0.05 with 0.5 nM and 1 nM BMP9, respectively) indicative of conserved and active canonical Smad signaling machinery (Supplementary Figure 3B). We subsequently tested the combined effect of treating cells with 1 nM BMP9 and either ALK1Fc or CFc on BRE reporter assay and found that treatment with ALK1Fc (10 μg/mL) completely abolished BMP9 signaling (Supplementary Figure 3C; BMP9+ALK1Fc) as evidenced by BRE-luc activity levels similar to that of cells without BMP9 treatment (Untreated). Treatment with BMP9+CFc (10 μg/mL) led to induction of BRE-luc activity that was similar to the level of BMP9 treatment alone (Supplementary Figure 3C; *p*-value < 0.05). Taken together, these results indicate that ALK1Fc blocks BMP9 signaling via ALK2 in PC-3MPro4 cells.

Moreover, ALK1Fc treatment, but not CFc, strongly reduced BMP9-induced cell proliferation in PC-3M-Pro4-Luc2 (*p* < 0.001 at day 4 comparing vehicle vs. BMP9 or ALK1Fc treatment, respectively; Figure 3B). This effect appeared to be specific (Supplementary Figure 3D) since treatment of PC-3M-Pro4-Luc2 cells with BMP9 in combination with an ALK2 small molecule kinase inhibitor (LDN193189, LDN) (Cuny et al., 2008; Shi et al., 2011) similarly resulted in the complete loss of BMP9-induced cell proliferation (Figure 3C). LDN treatment also blocked BMP9 stimulation of the BRE-luc reporter in PC-3M-Pro4-Luc2 cells (Supplementary Figure 3E). Together, these data indicate that ALK1Fc strongly reduces BMP9- induced proliferation in human prostate cancer cells.

### ALK1Fc inhibits ALDH1A1 expression *in vivo* and interferes with NOTCH signaling

**Figure 4:**
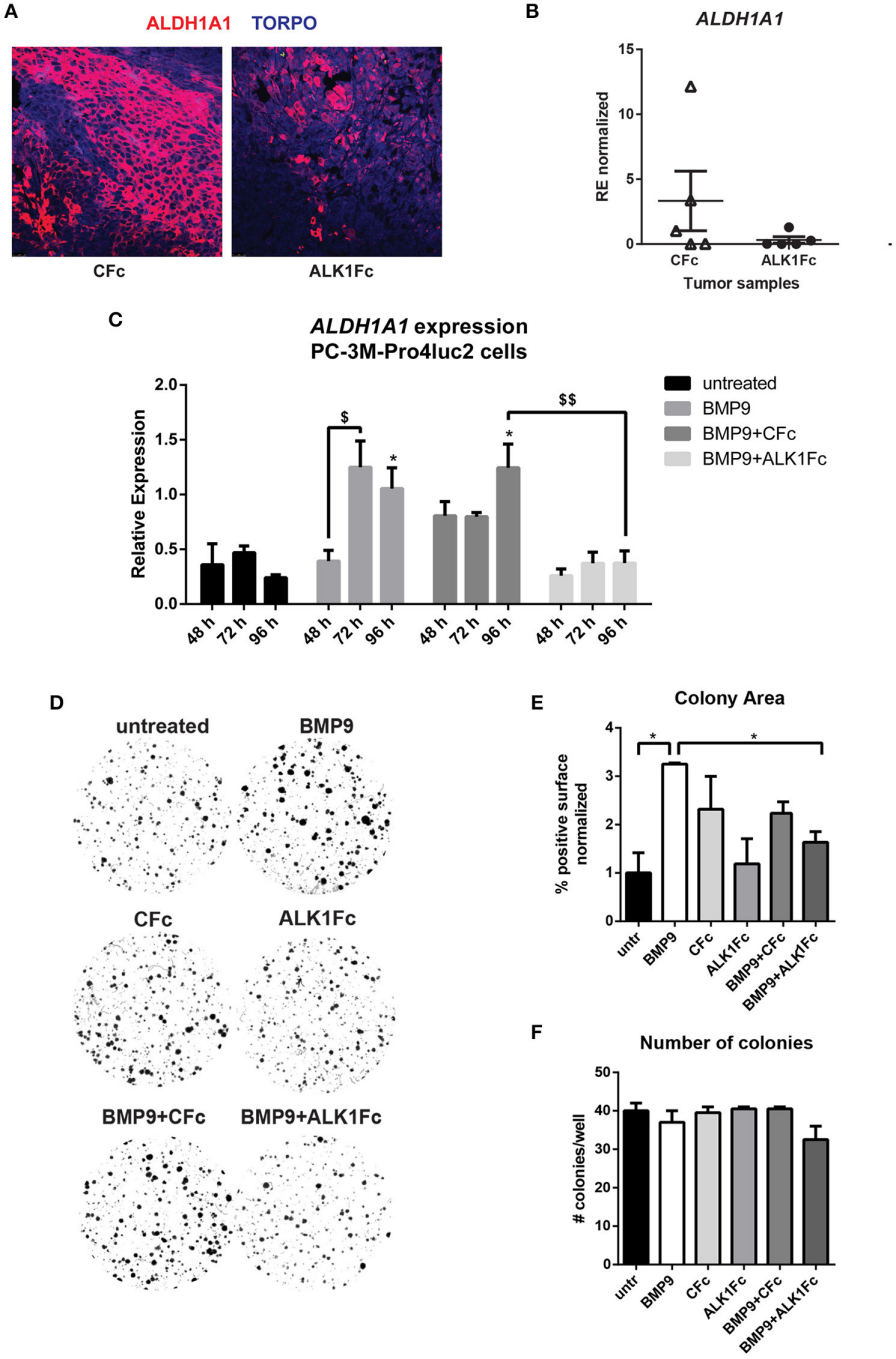
ALK1Fc treatment reduces ALDH1A1 expression. **(A)** Representative images of ALDH1A1 immunofluorescence in prostate tumor samples from ALK1Fc and CFc treated animals. ALDH1A1: red; TOPRO: blue. **(B)** Quantification of ALDH1A1 mRNA by Q-PCR in tumor samples of each group (*n* = 5 for CFc, *n* = 5 for ALK1Fc). **(C)** Expression of *ALDH1A1* in PC-3M-Pro4-Luc2 cells. Relative mRNA expression was measured by Q-PCR from cDNA obtained from PC-3M-Pro4-Luc2 cells treated with BMP9, BMP9+ALK1Fc, BMP9+CFc, for 48, 72, and 96 h. Values are normalized to β-actin expression. Error bars are ± SEM (*n* = 3). ^$^*P*-value < 0.05; ^$$^*P*-value < 0.01. **(D)** Clonogenic assay of PC-3M-Pro4-Luc2 cells. Low-density cultures (100 cells per well of 6 well plate) were stimulated with BMP9, CFc, ALK1Fc, BMP9+CFc, BMP9+ALK1Fc. Colony formation was assessed after 10 days by crystal violet staining. Representative images are shown. **(E,F)** Quantification of surface covered by crystal violet positive colonies (colony area) and colony number. Graph shows percentage of positive surface normalized per condition (average of three independent experiments). ^*^P-value < 0.05. Error bars indicate SEM.

Given its ability to reduce primary tumor burden and block BMP9-induced tumor cell proliferation *in vitro* and *in vivo*, we assessed the effects of ALK1Fc on the relative expression of *ALDH1A1*, a marker previously associated with cancer stem cell-like properties and poor patient prognosis (Li et al., 2010; Le Magnen et al., 2013). Treatment of prostate tumor bearing mice with ALK1Fc affected the number of ALDH1A1 positive cells in the prostate tumor tissues both at the protein (Figure 4A) and mRNA levels (Figure 4B). *In vitro* stimulation with BMP9 of the same cell line used to induce tumors in the xenograft mouse model confirmed that treatment with BMP9 or BMP9+CFc upregulates ALDH1A1 expression while BMP9+ALK1Fc treatment does not have any effect (Figure 4C).

We tested the effects of BMP9 treatment on the colony forming capacity of PC-3M-Pro4-Luc2 cells (Figure 4D) and found that it alters cell proliferation and strongly increasing the size of the colonies (Figure 4E, *p* < 0.05). However, BMP9 showed no effect on colony formation ability of PC-3M-Pro4-Luc2 since the total number of colonies formed with or without BMP9 treatment is similar (Figure 4F).

ALDH1A1 is known to be regulated by NOTCH signaling (Ginestier et al., 2007; Le Magnen et al., 2013; Zhao et al., 2014) and NOTCH1 plays a prominent role in prostate cancer cell proliferation and migration (Shou et al., 2001; Zhang et al., 2006; Leong and Gao, 2008; Bin Hafeez et al., 2009; Wang et al., 2010). Larrivee et al. have shown that ALK1 and NOTCH converge on common downstream pathways and that BMP9 treatment is sufficient to upregulate expression of the NOTCH pathway ligand JAG1 in HUVEC non-transformed cells (Larrivee et al., 2012).

**Figure 5:**
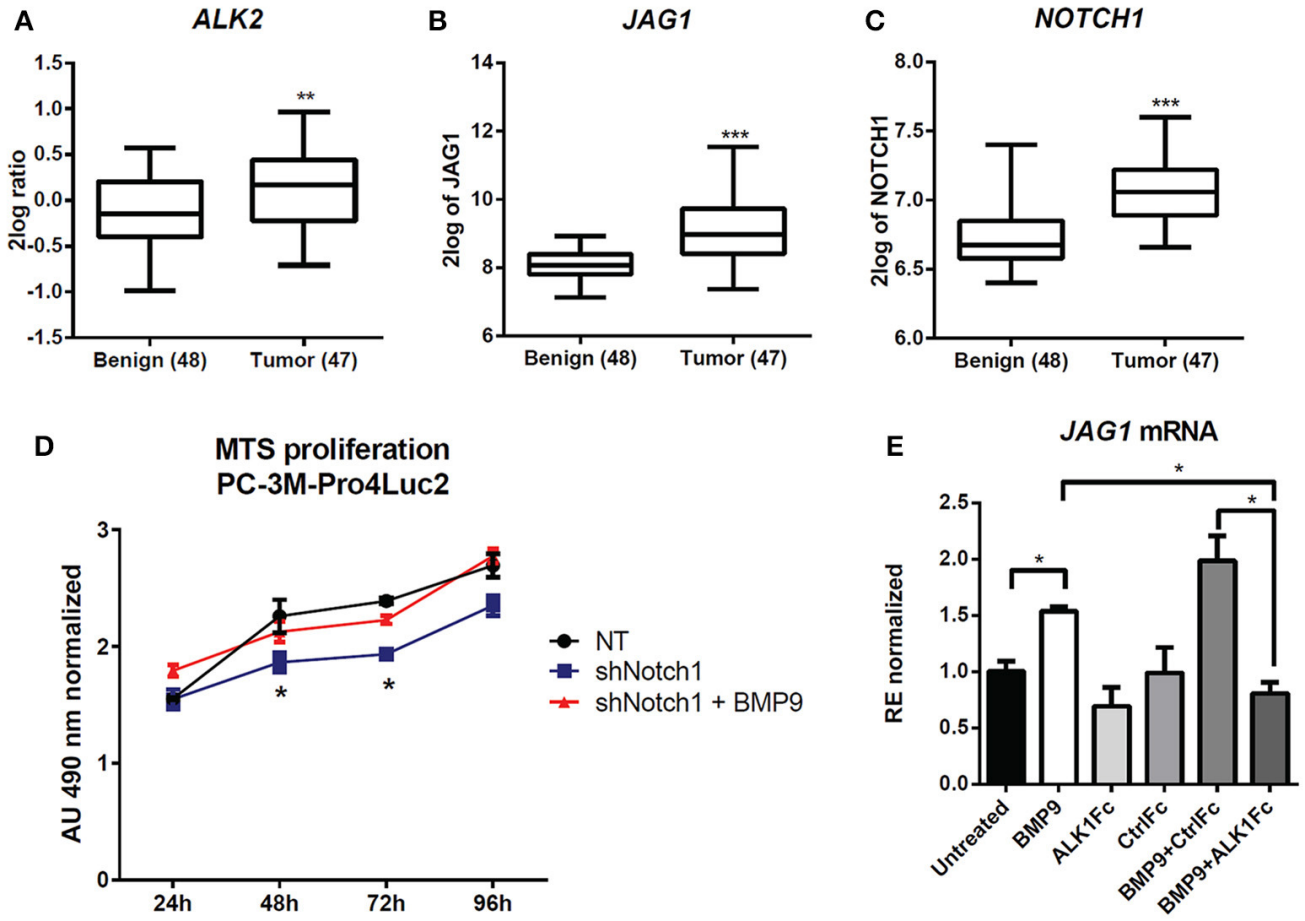
Effect of BMP9 and ALK1Fc on NOTCH signaling pathway. **(A–C)** Bioinformatic analysis of AMC OncoGenomics database (Sueltman transcript comparison) showing mRNA expression of *ALK2* **(A)** *JAG1* **(B)** and *NOTCH1* **(C)** in prostate tissues among benign prostate tissues (*n* = 48) vs. tumor tissues (*n* = 47). Values are expressed as 2log ratio tumor/benign. ^***^P-value < 0.001. **(D)** MTS assay (24, 48, 72, and 96 h) in PC-3M-Pro4-Luc2 cells transduced with short hairpin RNA against NOTCH1 (shNOTCH1) lentiviral vector or non-targeting (NT) shRNA vector (mock) and plated at low density. BMP9 (1 nM) was added once at cell seeding (*t* = 0). MTS absorbance was measured and values are normalized to the basal measurements *t* = 0 after cell seeding and treatments. Graph represents values for three independent experiments (*n* = 3). Error bars indicate SEM. ^*^P-value < 0.05. **(E)** Expression of *JAG1* in PC-3M-Pro4-Luc2 cells. Relative mRNA expression was measured by Q-PCR from cDNA obtained from PC-3M-Pro4-Luc2 cells treated with BMP9, ALK1Fc, CFc, BMP9+ALK1Fc or BMP9+CFc for 96 h. Values are normalized to β-actin expression. Error bars are ± SEM (*n* = 3). ^**^P-value < 0.01.

To assess the clinical relevance of crosstalk between BMP9/ALK2 signaling and NOTCH pathway activation in human prostate cancer, we performed bioinformatics analysis in 48 benign prostate tumors and 47 malignant prostate tumors (Borno et al., 2012) using R2 data mining platform (source: GEO ID: GSE29079). Transcript levels of *ALK2*, the NOTCH ligand *JAG1*, and *NOTCH1* were significantly higher in the malignant tumor group compared to the benign group (Figures 5A–C).

We targeted the expression of NOTCH1 in PC-3M-Pro4-Luc2 using a specific shRNA (shNOTCH1) and assessed resulting NOTCH1 levels by western blot and reporter assay (Supplementary Figures 4A,B). NOTCH1 knockdown led to a decreased proliferation rate compared to cells transduced with non-targeting shRNA lentivirus (*p* < 0.05 at 48 h and at 72 h) (Figure 5D). Notably, we observed that that shNOTCH1-cells display decreased levels of JAG1 mRNA (Supplementary Figure 4C) relative to control cells and that stimulation of shNOTCH1-cells with BMP9 rescued their proliferation rate (Figure 5D). To verify the effect of BMP9 on NOTCH signaling activation in our cancer model, we used qRT-PCR to quantify the expression of *JAG1* after BMP9 stimulation in presence of ALK1Fc or CFc. Our transcriptional analysis showed that BMP9 and BMP9+CFc induce mRNA expression of *JAG1* and that ALK1Fc treatment reduces this induction (Figure 5E). These data reinforce the hypothesis that the BMP9/ALK2 pathway can drive activation of NOTCH signaling linking two pathways that are associated with tumor progression in prostate cancer.

### ALK1Fc reduces tumor burden in the BM18 patient derived xenograft model

**Figure 6:**
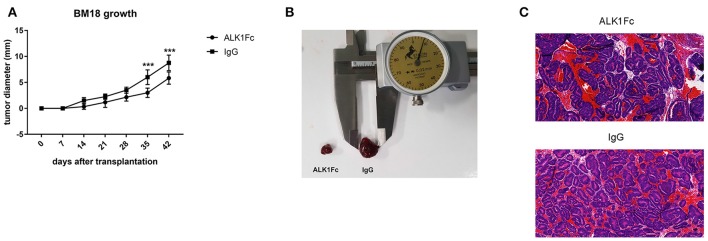
ALK1Fc reduces tumor burden in human prostate cancer xenograft BM18. Human PDX BM18 were transplanted subcutaneously in CB17 SCID mice (two tumors/animal) and treated either with ALK1Fc (*n* = 3) or IgG (*n* = 2). **(A)** Quantification of tumor burden by caliper measurement. Tumors treated with ALK1Fc *n* = 6; tumors treated with IgG *n* = 4. ^***^P < 0.01. Error bars indicate ± *SD*. **(B)** Representative images of BM18 tumor size after 5 weeks of treatment. **(C)** HE staining of BM18 after 5 weeks of treatment with ALK1Fc or IgG, left and right respectively.

While cell lines and mouse models are of great help in addressing biological questions, there is an increased need for personalized treatments and precision medicine based on screening of human material is becoming increasingly important. Therefore, we tested the antitumoral effect of ALK1Fc on the human patient derived xenograft (PDX) BM18. This PDX was derived from prostate cancer tissue harvested from femoral metastasis (McCulloch et al., 2005) and it is vitally maintained through serial passage in immunocompromised mice (Germann et al., 2012). We transplanted BM18 cells subcutaneously in severe combined immunodeficiency SCID mice and after 1 week the animals were treated with 10 mg/kg ALK1Fc or an IgG control once a week for an additional 5 weeks. Body weights of the mice were monitored weekly (Supplementary Figure 5) and the tumor growth was assessed by caliper measurement. We observed significant reduction in tumor burden upon ALK1Fc but not IgG administration (Figures 6A,B) and assessed the prostate epithelial phenotype of the tumors by the hematoxylin eosin (H&E) staining (Figure 6C).

## Discussion

In this study, we found that BMP9 has a tumor-promoting effect on human prostate cancer cells both *in vitro* and *in vivo*. We demonstrate that blocking BMP9 signaling with ALK1Fc efficiently diminishes prostate cancer cell proliferation and substantially attenuates tumor growth in both an orthotopic model of human prostate cancer and a prostate cancer derived PDX.

BMP9 was first identified in the liver (Song et al., 1995) and active forms are present in serum (Herrera et al., 2009). BMP9 is a ligand for ALK1 in endothelial cells (van Meeteren et al., 2012) and exerts stimulatory or inhibitory effects on endothelial cell growth and migration depending on the cellular context (David et al., 2007; Suzuki et al., 2010; Liao et al., 2017). Aberrant regulation of transforming growth factor-β (TGF-β) and BMP signaling often results in cancer progression (Siegel and Massague, 2003; Massagué, 2008). In particular, BMP ligands, such as BMP9 as well as BMP type I receptors (e.g., ALK1 and ALK2) have been associated with tumor angiogenesis and cancer progression. BMP9 signals through ALK2 in non-endothelial cells including those in ovarian epithelium, where it has been shown to promote proliferation of ovarian cancer cells (Herrera et al., 2009). Similarly, in hepatocellular carcinoma BMP9 has been reported to act as a factor that promotes cell proliferation and survival (Herrera et al., 2013). More recently, the BMP9/ALK2 axis has also been involved in erythroblast cancer cells proliferation (Kim et al., 2017). By contrast, studies have also highlighted the role of BMP9 in reducing breast cancer cell growth and metastasis (Wang et al., 2011, 2017; Ren et al., 2014a,b). Overall, the role of BMP9 and ALKs in promoting or suppressing different cancer types remains controversial and the effect of BMP9 on tumor promotion vs. tumor suppression is likely to be context and cancer-type specific. This provided the rationale for us to elucidate the role of BMP9 in prostate cancer, for which no information is available to our knowledge.

In our search of publicly available databases of human prostate cancer specimens we found that BMP9 was expressed at significantly higher levels in high risk prostate cancer patients compared to the low risk group and that ALK2 was significantly upregulated in malignant vs. benign tissue samples. These data are consistent with our model in which the tumor-promoting effects of BMP9 are mediated by ALK2. Additionally, microarray analysis of data from mouse prostate intraepithelial neoplasia (PIN) vs. invasive cancer in a multistage model of prostate carcinogenesis showed up regulation of ALK2 and BMP9 at the invasive stage in the stromal compartment (Bacac et al., 2006). These data, together with the anti-tumorigenic effect of ALK1Fc documented here, suggest a tumor-promoting role of BMP9 during prostate cancer progression.

Our *in vitro* findings strengthen the afore-mentioned expression data and suggest that BMP9 increases proliferation of human prostate cancer cells. Moreover, our studies with the ALK2 inhibitor LDN193189 support the notion that ALK2 is critically involved in mediating BMP9-induced proliferation in PC-3M-Pro4-Luc2 cells. As depicted in the Results and Supplementary Data sections, treatment with ALK1Fc or LDN193189 alone did not affect proliferation of human prostate cancer cells suggesting a paracrine effect of stroma-derived BMP9 on tumor cells.

We also used an orthotopic model of prostate cancer to demonstrate that ALK1Fc reduces prostate tumor burden and vascular density compared to the controls. Lectin distribution appeared to be less diffuse in ALK1Fc treated animals, suggesting an effect on vessel maintenance rather than angiogenesis. Strikingly, ALK1Fc treatment of tumor-bearing animals resulted in highly hypoxic tumors with a trend in decreased number of CD31+ tumor capillaries suggesting that ALK1Fc may block BMP9-induced neovascularization.

As expected, areas of tumor proliferation and apoptosis were found to be mutually exclusive in their distribution. Apoptotic regions overlapped with hypoxic areas, suggesting that blockade of BMP9 by ALK1Fc might have an effect on proliferation and apoptosis of human prostate cancer cells in addition to targeting vessel maintenance (Mitchell et al., 2010).

SMAD1 and SMAD5 are downstream intracellular effectors of BMP9 signaling and can directly interact with the JAG1 promoter and induce transcription of the NOTCH ligand JAG1 (Larrivee et al., 2012) following BMP9 treatment (Morikawa et al., 2011). Transcriptional analysis revealed that ALK1Fc systemically blocks the induction of JAG1 mRNA in the presence of BMP9 (Morikawa et al., 2011) supporting our hypothesis that the crosstalk between BMP9 and NOTCH signaling may have clinical implications in prostate cancer. Indeed, *in silico* analysis of a previously published dataset of human prostate cancer specimens confirms that both *NOTCH1* and *JAG1* are upregulated at the tumor stage (Borno et al., 2012). In particular, NOTCH signaling seems to promote epithelial-mesenchymal transition in prostate cancer cells (Zhang et al., 2017). Moreover, recent publication shows how NOTCH pathway inhibition antagonizes the growth and invasion of TMPERSS2-ERG positive prostate cancer cells (ERG overexpressing prostate tumor) (Kron et al., 2017) suggesting an important role of the cascade in tumor growth.

Interestingly, NOTCH activates ALDH1A1, an established marker for highly tumorigenic prostate cancer stem cell-like cells (Ginestier et al., 2007; Le Magnen et al., 2013; Zhao et al., 2014; Harris and Kerr, 2017). The ALDH1A1 subpopulation contributes to both tumor initiation and progression and when highly expressed in advanced-stage cancers correlates with poor survival in hormone-naïve patients (Le Magnen et al., 2013). Notably, we show here that ALK1Fc-treated tumors showed significant reduction of ALDH1A1. Taken together, these data suggest that ALK1Fc might potentially interfere with NOTCH signaling in the regulation of ALDH1A1.

Our conclusion that BMP9 promotes aggressive prostate cancer growth was further supported by our demonstration that administration of ALK1Fc inhibited the growth of BM18 PDX, an androgen-dependent bone metastatic prostate tumor. Importantly, these data confirm the ability of ALK1Fc to treat a tumor derived from human patient and open new perspectives in the clinical application of this ligand trap for the cure of prostate cancer.

Our findings provide novel information on the role of BMP9 in human prostate cancer and suggest the promising use of BMP9 targeting molecules for the treatment of tumor and supportive microenvironment in prostate cancer patients.

## Author contributions

LA, EZ, and SK: designed the work, acquired and interpreted the data and drafted the manuscript; PG interpreted the data and drafted and revised the manuscript; IK and JG acquired the data and revised the manuscript; MG, LH, GvdP, MS, GT interpreted the data and revised the manuscript; PtD conceived the work, interpreted the data and revised the manuscript; MK conceived the work, acquired and interpreted the data, drafted and revised the manuscript. All authors approved the version to be published and are accountable for all aspects of the work in ensuring that questions related to the accuracy or integrity of any part of the work are appropriately investigated and resolved.

### Conflict of interest statement

The authors declare that the research was conducted in the absence of any commercial or financial relationships that could be construed as a potential conflict of interest.
