# Supplementary material for: ALK1Fc Suppresses the Human Prostate Cancer Growth in in Vitro and in Vivo Preclinical Models
Source: Front Cell Dev Biol. 2017 Dec 5;5:104. doi: 10.3389/fcell.2017.00104 (PMC5723291; doi:10.3389/fcell.2017.00104)
Supplement: Supplementary file 1 [file Table1.DOCX]

| **GENE** | **SEQUENCE** | **SPECIES** |
| --- | --- | --- |
| ALK1_FW | ATGACCTCCCGCAACTCGA | Human |
| ALK1_RV | TAGAGGGAGCCGTGCTCGT | Human |
| ALK2_FW | TGCCTTCGAATAGTGCTGTC | Human |
| ALK2_RV | CATCAAGCTGATTGGTGCTC | Human |
| ALK4_FW | GCTCGAAGATGCAATTCTGG | Human |
| ALK4_RV | TTGGCATACCAACACTCTCG | Human |
| ALK5_FW | ACGGCGTTACAGTGTTTCTG | Human |
| ALK5_RV | GCACATACAAACGGCCTATCT | Human |
| β-ACTIN_FW | AATGTCGCGGAGGACTTTGATTGC | Human |
| β-ACTIN_RV | AGGATGGCAAGGGACTTCCTGTAAA | Human |
| ALDH1A1_FW | GATCCAGGGCCGTACAATAC | Human |
| ALDH1A1_RV | CAAATGAGCATAACCAACGG | Human |
| JAG1_FW | TGTGTAAACGCCAAATCCTG | Human |
| JAG1_RV | CGATAACCATTAACCAAATCCC | Human |
| GAPDH_FW | GACAGTCAGCCGCATCTTC | Human |
| GAPDH_RV | GCAACAATATCCACTTTACCAGAG | Human |

Supplementary Table I – Primer List
