# Supplementary material for: ALK1Fc Suppresses the Human Prostate Cancer Growth in in Vitro and in Vivo Preclinical Models
Source: Front Cell Dev Biol. 2017 Dec 5;5:104. doi: 10.3389/fcell.2017.00104 (PMC5723291; doi:10.3389/fcell.2017.00104)
Supplement: Supplementary file 2 [file DataSheet1.pdf]

Supplementary Material

ALK1Fc Suppresses the Human Prostate Cancer Growth in *In Vitro* And *In Vivo* Preclinical Models

Letizia Astrologo [†1], Eugenio Zoni[†1,2], Sofia Karkampouna[†1,3], Peter C. Gray[4], Irena Klima[1], Joël Grosjean[1], Marie Jose Goumans[3], Lukas J. A. C. Hawinkels[3,5], Gabri van der Pluijm[2], Martin Spahn[1], George N. Thalmann[1], Peter ten Dijke[3] and Marianna Kruithof-de Julio*[1,2,3]

*** Correspondence:**
Marianna Kruithof - de Julio, PhD

marianna.kruithofdejulio@dbmr.unibe.ch

Supplementary Figures

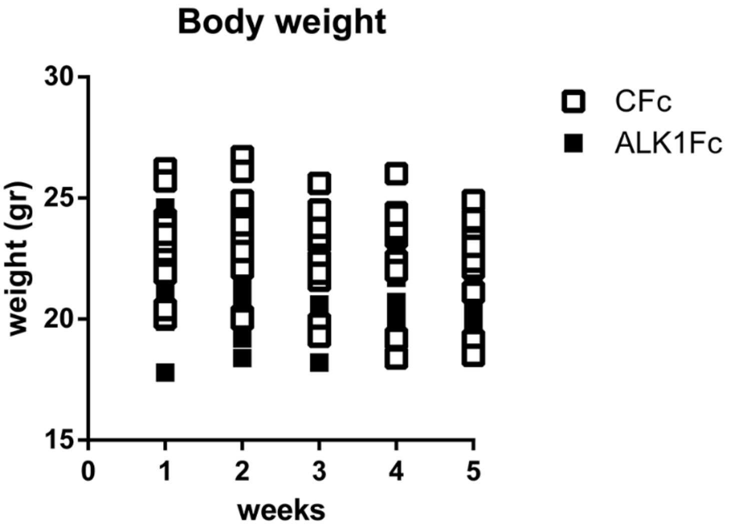

Supplementary Figure 1. Body weight of animals used in the orthotopic experiment. Body weight (grams) of all the animals measured weekly during the course of treatment with either CFc (n=6) or ALK1Fc (n=7).

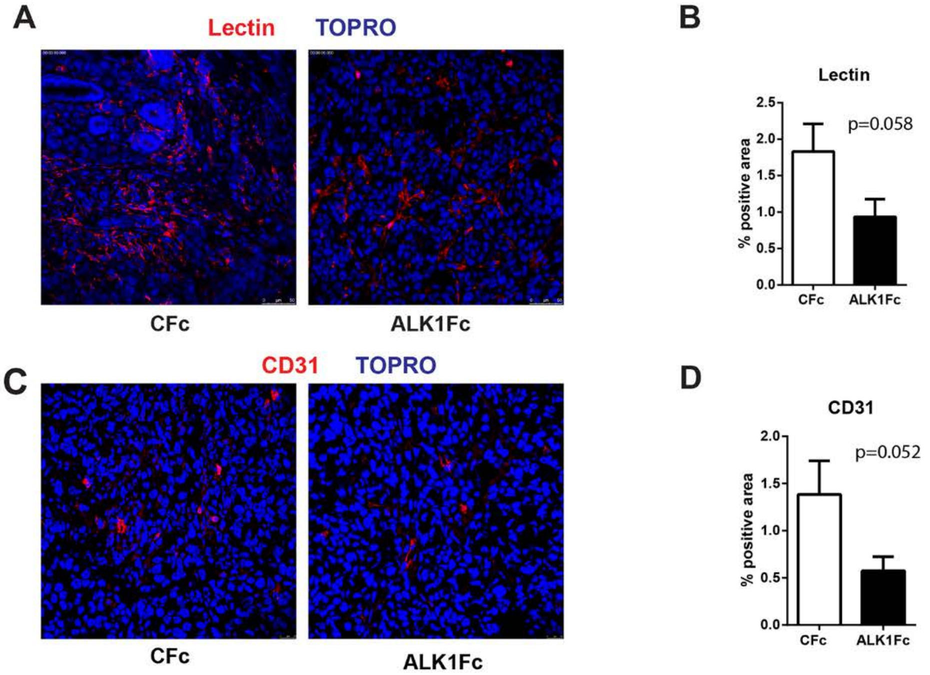

Supplementary Figure 2. Effect of ALK1Fc on vascular density *in vivo*. (A) Representative images of lectin detection in primary prostate tumor samples after perfusion with fluorescent-labelled lectin-Tomato (red). TOPRO (blue) marks the nuclei. Treatment groups: ALK1Fc or CFc. **(B)** Quantification of lectin positive surface area in all tumor samples of each group (n=6 for CFc, n=7 for ALK1Fc). **(C)** Representative images of CD31 (red) immunofluorescence staining in primary prostate tumor samples after 5 weeks of treatment with either ALK1Fc or CFc. **(D)** Quantification of CD31 positive surface area in all tumor samples of each group (n=6 for CFc, n=7 for ALK1Fc).

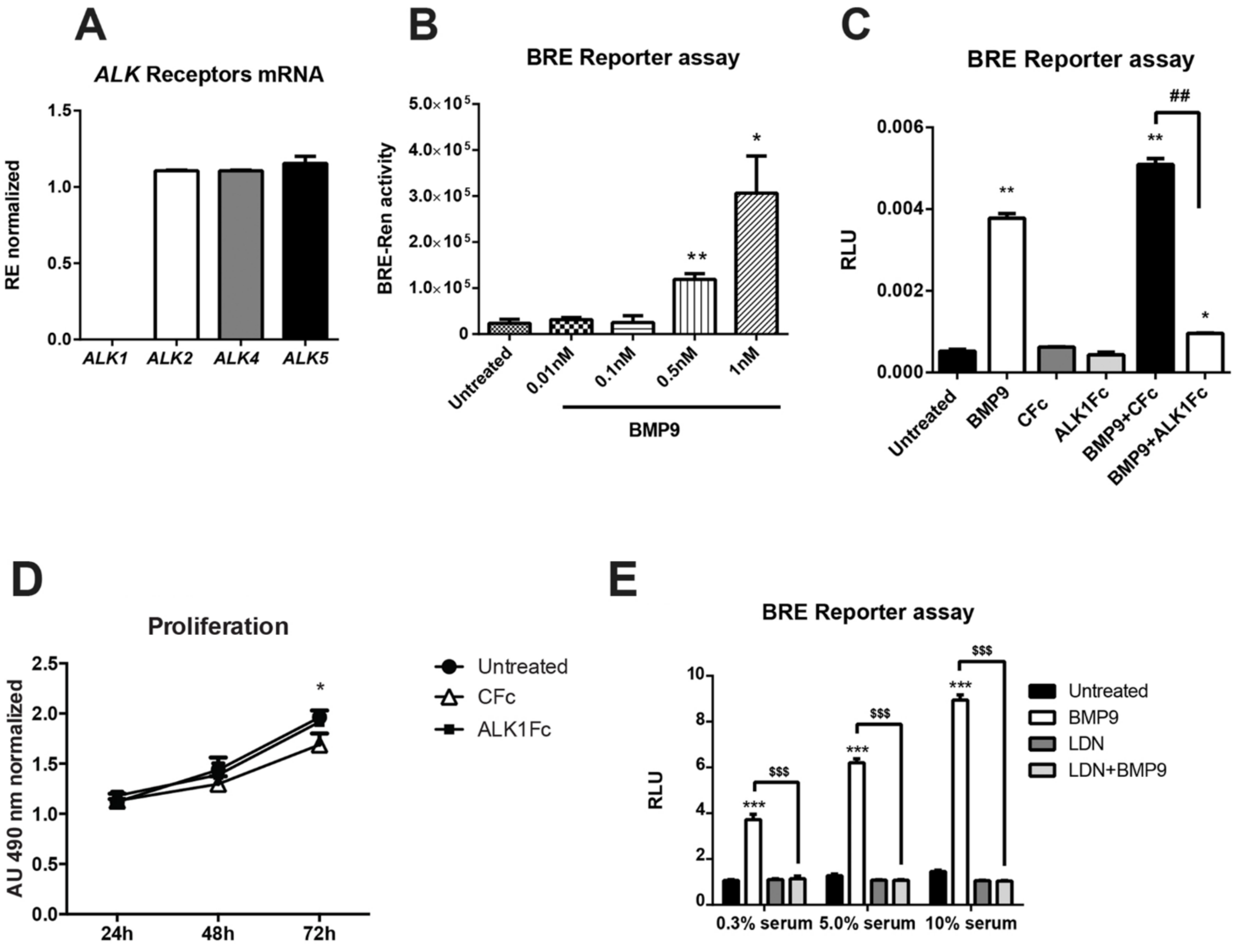

Supplementary Figure 3. Characterization of dose response for BMP9 with luciferase reporter **(A)** Endogenous expression of *ALK1*, *ALK2*, *ALK4* and *ALK5* receptors in PC-3M-Pro4-Luc2cells (mRNA level). Relative expression levels normalized to β-actin are shown. Error bars indicate +/-SD. **(B)** Dose dependent response of PC-3M-Pro4 cells to recombinant BMP9 (0.01, 0.1, 0.5, 1 nM). Downstream activation of BMP signaling was tested by transfection of BRE-renilla construct and measured by reporter activity assay. Graph represents values from three independent experiments; error bars indicate ± SEM (n=3). P value < 0.05 (*) and P value < 0.01 (**). **(C)** BRE reporter luciferase (BREluc) assay; Inhibitory concentration of ALK1Fc or CFc (10μg/ml) was determined in cells stimulated with 1nM BMP9. Graph represents values from two independent experiments; error bars indicate ± SEM (n=2). P value < 0.05 (*) and P value < 0.01 (**) compared to "untreated" control. P value < 0.01 (##) **(D)** MTS assay (24, 48, 72 hours) was performed in PC-3M-Pro4-Luc2 human prostate cancer cell line treated with recombinant ALK1Fc (10μg/ml) or CFc (10μg/ml). Accumulation of MTS was measured based on absorbance at 490 nm. Values are normalized to the basal measurements at 24 hours after cell seeding and treatments. Graph represents values from two independent experiments (n=2). Error bars indicate ±SEM. (**E**) BMP promoter assay (BRE-luciferase). PC-3M-Pro4 were seeded and transfected with BRE-luc and renilla plasmid DNA. After 24 hours the medium was replaced with 0.3%, 5%, 10% FCII serum containing media and treated with BMP9

(1nM), LDN193189 (LDN, 120nM) and BMP9+LDN. Luc and Ren values were measured 24 hours after treatment. RLU ratio values are shown (Luc/Ren). Error bars indicate ±SD.

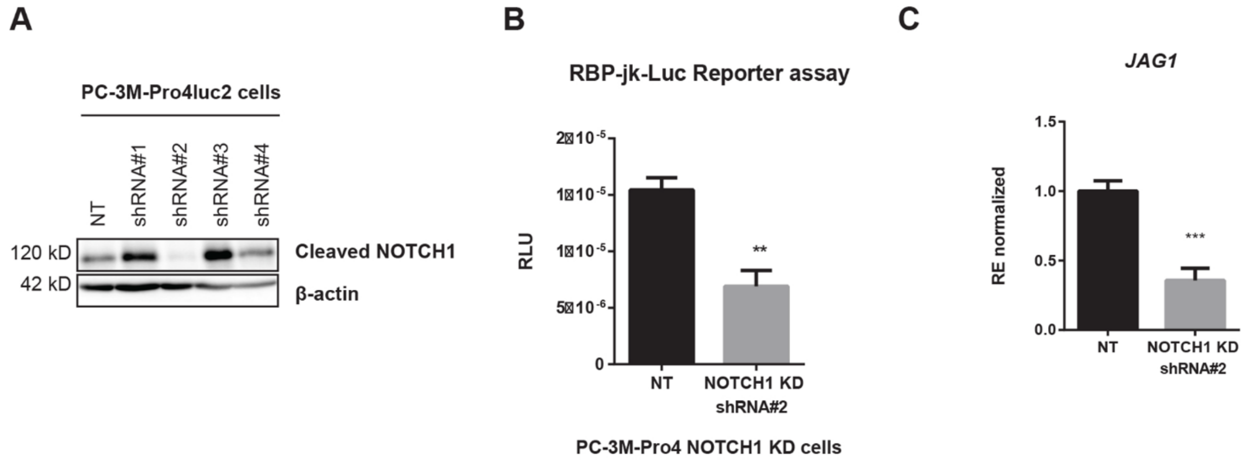

Supplementary Figure 4. Characterization of NOTCH1 knockdown. (A) Western blotting for NOTCH1 protein as validation of lentiviral shRNA-mediated knockdown of NOTCH1 intracellular domain (cleaved) in PC-3M-Pro4-Luc2 cell. Based on the downregulation of cleaved NOTCH1 observed after lentiviral transduction and puromycin selection, the stable line expressing the shRNA #2 construct was selected for further experiments. NT; non-targeting shRNA lentiviral mediated transduction. β-actin was used as loading control. **(B)** NOTCH transcription factor RBP-Jk-luciferase reporter assay in non-targeting (NT) and NOTCH1 (shRNA#2) knockdown (KD) PC-3M-Pro4-Luc2 cells. RLU: relative luciferase units (signal of luciferase normalized to renilla values). n=3, P value < 0.01 (**) **(C)** Q-PCR for *JAG1* mRNA levels in non-targeting (NT) and NOTCH1 (shRNA#2) knockdown (KD) PC-3M-Pro4-Luc2 cells. Fold over the value of NT is represented. Error bars indicate ±SD. P value < 0.001 (***).

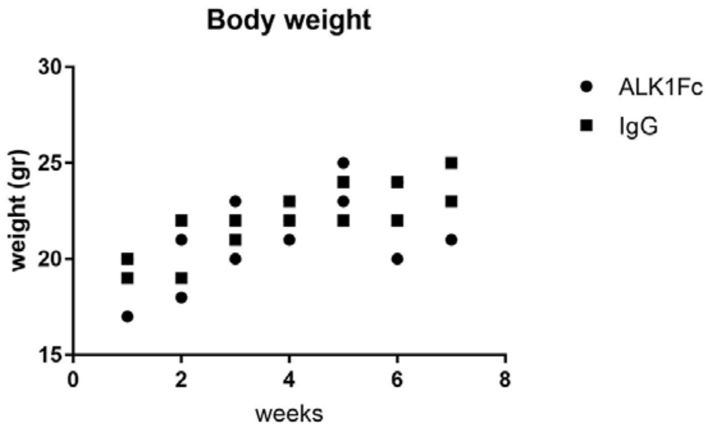

Supplementary Figure 5. Body weight of animals used in the BM18 experiment. Body weight (grams) of all the animals measured weekly during the course of treatment with either IgG (n=2) or ALK1Fc (n=3).
